# Primary Low-Grade Peritoneal Serous Carcinoma in a Woman With Psammoma Bodies on Cervicovaginal Cytology

**DOI:** 10.7759/cureus.61056

**Published:** 2024-05-25

**Authors:** Eduardo Gonzalez-Bosquet, Marta Avella Marcos, Carlota Rovira

**Affiliations:** 1 Obstetrics and Gynecology, Hospital Sant Joan de Déu, University of Barcelona, Barcelona, ESP; 2 Pathology, Hospital Sant Joan de Déu, University of Barcelona, Barcelona, ESP

**Keywords:** primary peritoneal, laparoscopy, low-grade serous carcinoma, cervicovaginal cytology, psammoma bodies

## Abstract

Psammoma bodies in cervicovaginal cytology are a rare finding associated with malignant tumours. A 62-year-old woman was referred to our centre for cytology with nuclear atypia and psammomatous bodies suspicious of malignancy. A complete gynaecological examination was performed including colposcopy and ultrasound without significant changes. Hysteroscopy was performed to detect endometrial or endocervical malignancy, endometrial biopsy showed psammoma bodies and atrophic endometrium. Endocervical and cervical biopsies were negative for malignancy. Cervicovaginal cytology and human papillomavirus (HPV) testing were repeated. The result was suggestive of adenocarcinoma and negative for HPV. Laparoscopic hysterectomy with bilateral salpingo-oophorectomy was indicated due to two cervicovaginal cytologies with suspicion of malignancy. Low-grade peritoneal serous carcinoma was diagnosed on the surface of the uterus, ovaries and peritoneum. A second laparoscopy was performed to exclude other pelvic or abdominal lesions, and disease was found in the peritoneum of the pelvis, abdomen and omentum. Adjuvant treatment with six cycles of carboplatin and paclitaxel was indicated. Psammoma bodies in cervicovaginal cytology are a rare clinical situation, and it is mandatory to exclude malignancy.

## Introduction

Primary peritoneal carcinoma (PPC) is a rare neoplasm that diffusely affects the peritoneal surface with minimal ovarian involvement [1-4]. PPC is indistinguishable from ovarian cancer and has similar clinical behaviour, patterns of spread, response to treatment and survival rates [5-7]. Most of the literature evaluating PPC has focused on patients with high-grade serous tumours [8]. Psammoma bodies in cervical smears are associated with malignancy, particularly ovarian cancer [9].

We present the case of a woman who had psammoma bodies on her cervical smears and was diagnosed with primary low-grade peritoneal serous carcinoma.

## Case presentation

**Figure 1:**
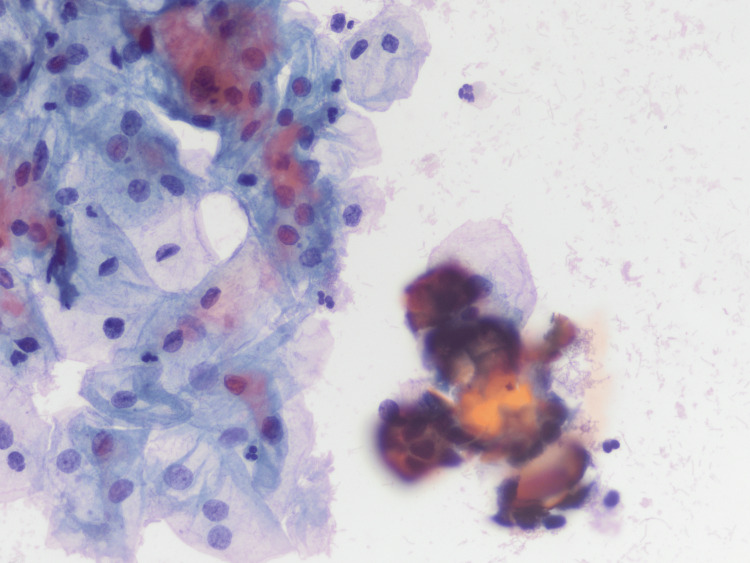
Gynaecologic pap: cervical cytology with glandular cells with low atypia and occasional calcification (PAPX400).

A 62-year-old woman with a significant history of hypertension, type 2 diabetes mellitus and a BMI of 31. She was referred to our centre for cytology with nuclear atypia and psammomatous bodies (PB) suspicious of malignancy (Figure 1).

A complete gynaecological examination was performed including colposcopy and ultrasound. Colposcopy showed a type 3 transformation zone without any lesion in the cervix and vagina. The vaginal ultrasound did not reveal uterus or ovarian alterations, and the endometrium was 2 mm thick. A new cervical smear showed glandular changes suggestive of adenocarcinoma, and endocervical curettage and HPV testing were negative.

Hysteroscopy was performed to detect endometrial or endocervical malignancy, with negative results. Multiple endometrial, endocervical and cervical biopsies were performed. Endometrial biopsy showed psammoma bodies and atrophic endometrium, a benign endocervical polyp was removed, endocervical curettage revealed a low-grade intraepithelial lesion (LSIL), and cervical biopsy was negative (no atypia).

Cervicovaginal cytology and HPV testing were repeated a month after hysteroscopy. The cervical smear was suggestive of adenocarcinoma and the HPV result was negative for infection.

**Figure 2:**
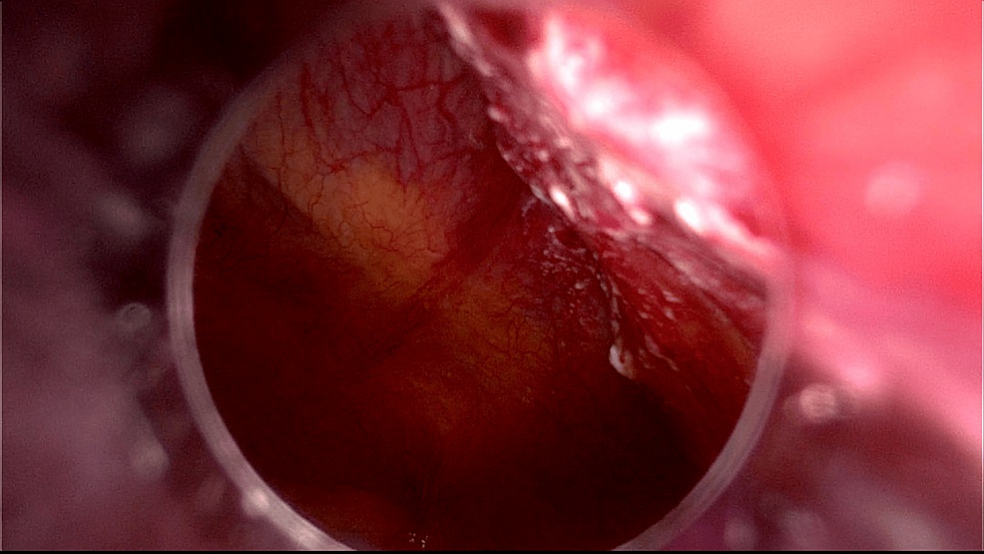
An abnormal white area on the peritoneal surface near the umbilical trocar can be seen through a laparoscopic view.

**Figure 3:**
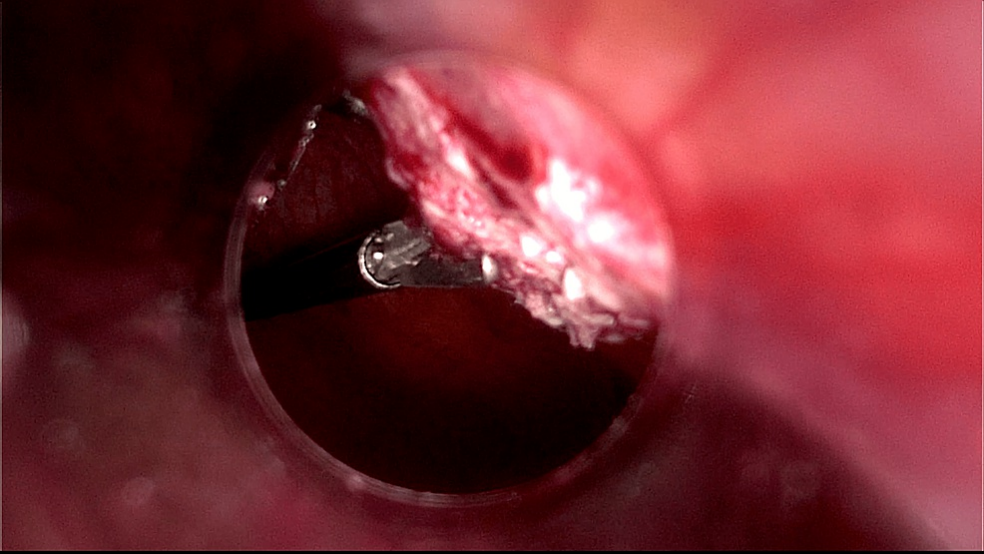
A laparoscopic view of the biopsy performed on peritoneum.

Due to two cervical smears suspected of malignancy, a laparoscopic hysterectomy with bilateral salpingo-oophorectomy was recommended in May 2022. The case was not presented in the tumour board because the risk of malignancy was thought to be low since all previously performed biopsies and imaging tests were negative. During surgery, a white area of 2-3 cm on the peritoneal surface near the umbilical trocar was noted on the examination of the abdominal cavity (Figure 2) and a biopsy was performed (Figure 3).

The pelvis had a green fluid that was aspirated for cytological examination. Despite being normal, the uterus and ovaries showed certain adhesions to both the fallopian tubes and the peritoneum. A hysterectomy was performed without any complications, and the uterus, ovaries and fallopian tubes were removed vaginally.

**Figure 4:**
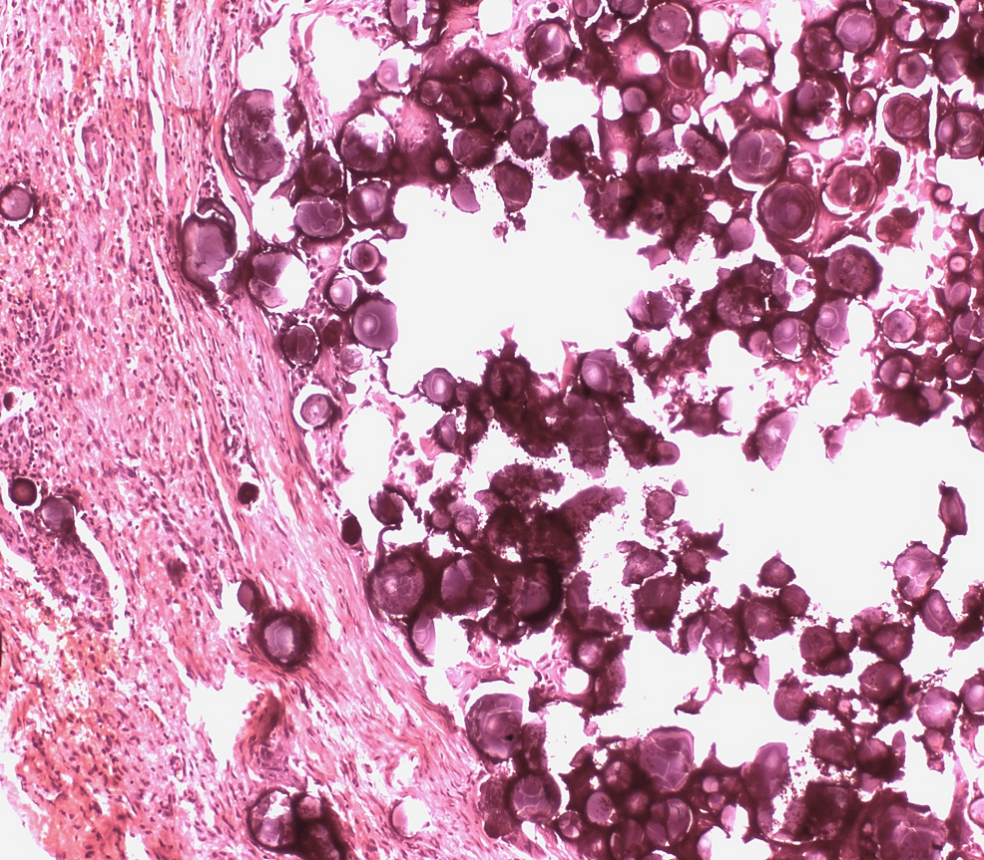
Dense fibrous tissue containing psammomatous calcifications suggestive of low-grade peritoneal serous carcinoma (H&E).

The postoperative period was uneventful and the patient was discharged two days after surgery. Histological examination showed proliferation of glandular epithelium with papillary structures and mild atypia, areas of fibrosis and multiple psammoma bodies on the surface of the uterus, ovaries, fallopian tubes and in the peritoneal biopsy without invasion, suggestive of low-grade peritoneal serous carcinoma (Figure 4).

**Figure 5:**
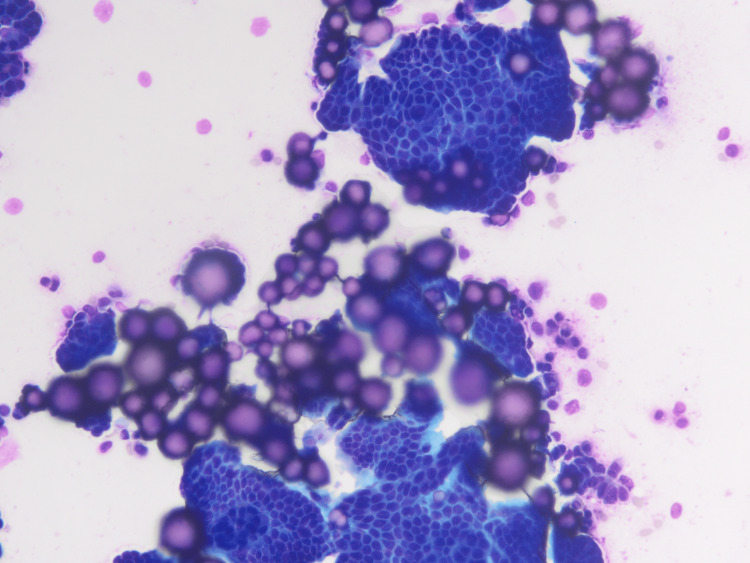
Serous fluid cytopathology with highly cellular smears with mesothelial cells and numerous psammoma bodies (DQ and pap x400).

Cytologic study of the peritoneal fluid shows atypical cells with psammoma bodies suggestive of malignancy (Figure 5).

The case was presented to the tumour board and magnetic resonance imaging (MRI) was performed to rule out other pelvic lesions. Small images measuring 9 to 4 mm were observed in the pelvis MRI. Ca 125 and HE4 tumour markers were negative (17 U/mL and 55 pmol/L). A new laparoscopy was carried out and 4-mm nodules were resected. An omentectomy and several peritoneal biopsies were also performed, but no tumour lesions remained. Lymphadenectomy was not performed because no suspicious lymph nodes were observed on MRI.

Histological examination of all biopsies revealed a similar lesion to that found in the previous biopsies, suggesting a low-grade peritoneal serous carcinoma. The case was presented to the tumour board and classified as stage IIIC cancer due to the involvement of the omentum and peritoneal. It was recommended to receive six cycles of carboplatin and paclitaxel as adjuvant treatment. The treatment was finished in April 2023. The patient has been disease-free 19 months after diagnosis.

## Discussion

It is uncommon to find psammoma bodies in cervical smears [9]. In a large study of 234,318 cervicovaginal smears, only seven smears contained psammoma bodies (prevalence 0.00003)[10]. Four of the seven were associated with cancer: two papillary serous carcinomas of the endometrium, one serous cystadenocarcinoma of the ovary and one papillary serous carcinoma of the peritoneum [10], the latter being similar to our case.

Other studies have found approximately 61-50% of cases of women with PB in cervicovaginal smears are associated with benign conditions, such as endosalpingiosis [9-11]. Other authors associate the presence of psammoma bodies with endometrial changes due to the use of a combined oral contraceptive agent [12]. Women with PB and benign conditions are younger (mean age 42.7 years), do not have symptoms suggestive of malignancy and do not have highly atypical or malignant appearing glandular cells [11]. Women with PB and underlying malignancy were older (mean age 56 years) and postmenopausal, in our case 62 years, and had highly atypical or malignant glandular cells, as in our case.

In our case, the presence of psammoma bodies in the endometrial biopsy suggests that these structures passed through the tube and endometrial cavity to the cervix.

We found few studies in the literature regarding low-grade serous PPC, Schmeler et al. described one of the largest series with 53 cases [8], most cases were diagnosed with stage III as in our case. HE4 and CA125 levels were not significantly elevated in low-grade PPC [13]. The primary treatment was surgical cytoreduction, which was achieved in 46 patients (86.8%) in Schmeler et al.’s series [8], an optimal surgical cytoreduction was also achieved in our case. Most patients (88%) received systemic therapy, with a combination of taxane and platinum [8-14], as in our case.

It is also noteworthy that 69.3% of women with low-grade PPC were overweight, as in our case, with BMI of 31, compared to women with low-grade serous ovarian cancer [15].

## Conclusions

The finding of psammoma bodies in cervicovaginal cytology is a rare clinical situation and it is imperative to rule out malignancy especially when associated with glandular cells of highly atypical or malignant appearance. Ovarian, endometrial or primary peritoneal cancer must be excluded.
